# Sequestration and Destruction of Rinderpest Virus–Containing Material 10 Years after Eradication

**DOI:** 10.3201/eid2809.220297

**Published:** 2022-09

**Authors:** Christine M. Budke, Dirk U. Pfeiffer, Bryony A. Jones, Guillaume Fournié, Younjung Kim, Mariana Marrana, Heather L. Simmons

**Affiliations:** Texas A&M University, College Station, Texas, USA (C.M. Budke);; Texas A&M AgriLife Research, College Station (C.M. Budke, H.L. Simmons);; Royal Veterinary College, London, UK (D.U. Pfeiffer, G. Fournié);; City University of Hong Kong, Hong Kong (D.U. Pfeiffer, Y. Kim);; Animal and Plant Health Agency, Weybridge, UK (B.A. Jones);; University of Sussex, Brighton, UK (Y. Kim);; World Organisation for Animal Health, Paris, France (M. Marrana)

**Keywords:** rinderpest virus, morbillivirus, viruses, cattle, eradication, biosecurity

## Abstract

In 2021, the world marked 10 years free from rinderpest. The United Nations Food and Agriculture Organization and World Organisation for Animal Health have since made great strides in consolidating, sequencing, and destroying stocks of rinderpest virus–containing material, currently kept by only 14 known institutions. This progress must continue.

In 2011, ten years after the last confirmed outbreak, the Food and Agriculture Organization of the United Nations (FAO) and the World Organisation for Animal Health (WOAH, formerly OIE) jointly declared global freedom from rinderpest. Rinderpest, also known as cattle plague, is only the second infectious disease eradicated from the world, smallpox being the first. Over the 10 years since eradication, the main goal of the Rinderpest Post-Eradication Programme (https://www.woah.org/en/disease/rinderpest) has been to track and reduce global stocks of rinderpest virus–containing material (RVCM). 

RVCM comprises field and laboratory strains of rinderpest virus; vaccine strains of rinderpest virus, including valid and expired vaccine stocks; tissues, serum, and other clinical material from infected or suspect animals; diagnostic material containing or encoding live virus; recombinant morbilliviruses (segmented or nonsegmented) containing unique rinderpest virus nucleic acid or amino acid sequences; and full-length genomic material, including from virus RNA and cDNA copies of virus RNA. Subgenomic fragments of morbillivirus nucleic acid not capable of incorporation into a replicating morbillivirus or morbillivirus-like viruses are not considered RVCM. 

Accounting for remaining RVCM is critical to limit the risk for reintroducing the pathogen by intentional or inadvertent release from a laboratory (*1*). In support of this effort, in 2015, FAO and WOAH started the Sequence and Destroy project, which enabled whole-genome sequencing of rinderpest virus (RPV) isolates before their destruction. Participating institutes were expected to deposit the genome sequences into publicly accessible databases. In addition, FAO has provided hands-on assistance and remote support to destroy viral stocks in Africa and Asia and led organization of >5 global and regional advocacy meetings. During June–October 2021, a review was conducted to mark progress towards RVCM sequestration and destruction 10 years after eradication. We report the main findings of this review. 

## The Study

In 2011, a total of 150 countries were surveyed regarding their RVCM stocks (*2*). At that time, 35 countries (44 laboratories) reported keeping RVCM. In 2013, WOAH began annual surveys of institutes keeping RVCM. In 2013, a total of 23 countries reported keeping RVCM; 13 kept live virulent virus and 19 live-attenuated virus in the form of vaccine (n = 17) or seedstock (n = 17), and 9 countries kept both virulent virus and vaccine. Because FAO and WOAH worked with members to eliminate or transfer RVCM stocks, the number of countries keeping RVCM had decreased to 12 (14 institutes) as of 2021 (Figure). In addition, FAO/WOAH designated some of these institutes either category A or B or dual-category rinderpest-holding facilities (RHFs) (https://www.oie.int/en/disease/rinderpest/#ui-id-3). Category A RHFs are designated for storing RVCM, excluding vaccine stocks; category B RHFs are approved for storing only manufactured vaccines and materials for their production. 

**Figure:**
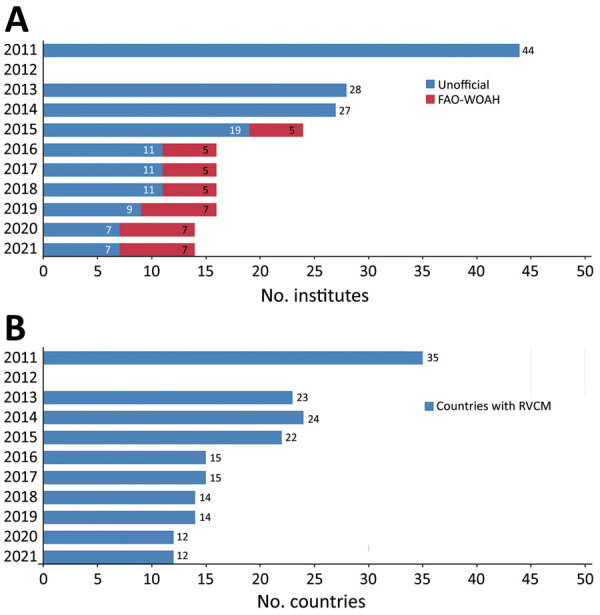
Number of institutes (A) and countries (B) keeping RVCM, by year, 2011 and 2013–2021. Data from 2011 are based on a single study, whereas data for 2013–2021 are based on WOAH country reports and institute director interviews (2021). FAO, Food and Agriculture Organization of the United Nations; RVCM, rinderpest virus–containing material; WOAH, World Organisation for Animal Health.

To confirm that no relevant findings unknown to WOAH had been published by research groups or laboratories, we reviewed the scientific literature to identify any publications about rinderpest virus research undertaken since 2011. A search of 21 databases identified 623 unique publications of which we evaluated 17 at the full-text level (Appendix Table 1). The search identified no institutes conducting work with RVCM not already known to WOAH. Nine (53%) of 17 reviewed studies were conducted in facilities that are FAO/WOAH-designated RHFs; 4/17 were published in 2011. Besides genome sequencing data, the main finding from recent research was that vaccination of cattle with peste des petits ruminants virus (PPRV) does not provide protective immunity against RPV (*3*), leading to the decision to maintain and even expand global contingency stocks of RPV vaccine (Appendix Table2). 

Members of the study team contacted a representative from each institute known by WOAH to keep RVCM as of August 2021 to arrange an interview to discuss current and historic RVCM stocks and laboratory biosecurity. Interviews were conducted remotely and accompanied by completion of a structured questionnaire. All institutes keeping RVCM, except for 1 located in the Middle East, responded to the request for an interview. Because of logistical difficulties and COVID-19–related challenges, interviews were not conducted with institutes in 2 countries in Europe. Therefore, during August 8–September 17, 2021, interviews proceeded with 11 of the 14 institutes known to keep RVCM. Several of the institute directors contacted were not familiar with the specific content of their RVCM stocks and indicated that these materials were simply in storage, which is concerning because of the critical nature of these materials. At present, Africa is the only region actively attempting to consolidate its RVCM into a single facility. 

According to Resolution 18, passed in 2011 during the 79th general session of the World Assembly of WOAH Delegates: “Rinderpest virus-containing material that is not in an approved BSL3 [Biosecurity Level 3] facility shall be destroyed by a validated process or transferred to an approved BSL3 facility.” Biosecurity levels for institutes keeping RVCM during 2011–2021 ranged from BSL2 to BSL4 (Table) meaning some institutes still do not meet this requirement; continued efforts are therefore needed. Three category B RHFs have actively contributed to the global rinderpest vaccine reserve. One institute in Europe keeps a rinderpest RBOK (Muguga-modification of the Kabate-0-strain) vaccine seed bank sufficient to produce ≈800,000 doses. One institute in the WOAH Asia and the Pacific region biannually produces a total reserve of ≈772,000 doses of LA-AKO (master seed virus) strain vaccine. One institute in Africa has a historic reserve of ≈959,000 doses of RBOK vaccine. Three RHFs have participated in sequence and destroy projects, and 2 more have initiated the approval process for sequence and destroy projects from the FAO/WOAH rinderpest secretariat. 

**Table:** Regional institutes with rinderpest virus–containing material, by biosafety level and type, for 2013 and 2021*

Category	Africa	Asia, Far East, and Oceania	Europe	Middle East	The Americas	World
Biologic safety level						
2	3/0	1/2	0/0	1/1	1/0	6/3
2+	0/0	0/1	0/0	0/0	0/0	0/1
3	4/1	9/3	3/3	0/0	2/1	18/8
3+	0/0	0/0	1/1	0/0	0/0	1/1
4	0/1	0/0	2/0	0/0	0/0	2/1
Unknown	1/0	0/0	0/0	0/0	0/0	1/0
Type						
A	0/0	0/1	0/1	0/0	0/1	0/3
B	0/0	0/1	0/0	0/0	0/0	0/1
A/B	0/1	0/1	0/1	0/0	0/0	0/3
Unofficial	8/1	10/3	6/2	1/1	3/0	28/7
Overall	8/2	10/6	6/4	1/1	3/1	28/14

*Values are given as 2013/2021 numbers.

During recent genomic analysis of PPRV isolates held at an FAO/WOAH–designated RHF, 1 sample was found to contain a sequence that aligned with RPV in addition to PPRV sequences. A traceback investigation found that this sample, obtained from the field by another institute in the early 1970s, appears to have been destroyed. All materials derived from the original stock before the contaminated sample was identified were uncontaminated, but those derived from the contaminated stock were RPV-contaminated, so contamination appears to have occurred inside the institute, from an unknown source, but likely during a period in the late 1990s when both RPV and PPRV were being manipulated concurrently at the institute. All contaminated samples were destroyed. After examining all other samples being manipulated during the same period, the institute concluded that no others were contaminated. Records indicated that the institute had not shared this sample with other facilities and that it would thereafter screen all PPRV samples by PCR for RPV before sharing them. No contaminated samples had escaped containment and all processes to secure stocks appeared to be working well. Risks associated with remaining global stocks are being evaluated and will be presented in a future publication.

## Conclusions

We document discovery of RPV-contaminated PPRV samples; our findings suggest that because of risk for cross-contamination, other laboratories should take precautions with samples manipulated alongside RPV, especially PPRV. Although progress is being made in consolidating RVCM stocks, 2 of 6 nonapproved institutes known to keep RVCM stockpiles have indicated no plans to destroy or transfer them to an FAO/WOAH RHF. Therefore, in spite of the progress, much work remains. Current FAO/WOAH strategy is to continue removing RVCM from nonapproved laboratories and advocating for reduced RVCM stocks in FAO/WOAH-designated RHFs. Ultimately, the only remaining RCVM materials should be manufactured vaccines and materials for vaccine production and diagnostics. 

Appendix Additional information from a study of sequestration and destruction of rinderpest virus–containing material
