# Correction: Vol. 26, No. 9

**DOI:** 10.3201/eid2810.C32810

**Published:** 2022-10

**Keywords:** errata, erratum, correction

The descriptions in the Acknowledgments have been expanded and updated for Sequestration and Destruction of Rinderpest Virus–Containing Material 10 Years after Eradication (C.M. Budke et al.). The article has been corrected online (https://wwwnc.cdc.gov/EID/article/28/9/22-0297_article).

