# Supplementary material for: Sequestration and Destruction of Rinderpest Virus–Containing Material 10 Years after Eradication
Source: Emerg Infect Dis. 2022 Sep;28(9):1895–8. doi: 10.3201/eid2809.220297 (PMC9423901; doi:10.3201/eid2809.220297)
Supplement: Appendix — Additional information from a study of sequestration and destruction of rinderpest virus–containing material [file 22-0297-Techapp-s1.pdf]

Article DOI: https://doi.org/10.3201/eid2809.220297

Sequestration and Destruction of Rinderpest Virus–Containing Material 10 Years after Eradication

Appendix

Appendix Table 1. Search terms used in the literature review to identify publications related to rinderpest virus research undertaken since 2011, among 21 databases searched

Search terms	Results
Global Health	
1910–2021 week 26	
exp Rinderpest morbillivirus/ or exp rinderpest/ or rinderpest*.ti,ab	432
Limited to 2011–2022	81
EMBASE	
OVID interface	
1974–2021 June 30	
exp Rinderpest virus/ or exp rinderpest/ or exp Cattle plague virus/ or rinderpest*.ti,ab.	829
Limited to 2011–2022	192
CAB Abstracts	
OVID interface	
1910–2021 Week 26	
exp Rinderpest morbillivirus/ or exp rinderpest/ or rinderpest*.ti,ab.	4,340
Limited to 2011–2022	274
International Pharmaceutical Abstracts	
OVID interface	
1970-June 2021	
Rinderpest*.ti,ab.	1
JBI EBP	
OVID interface	
current to June 23, 2021	
Rinderpest*.ti,ab.	1
Northern Light Life Sciences Conference Abstracts	
OVID interface	
2010–2021 Week 25	
Rinderpest*.ti,ab.	6
Limited to 2011–2022	5
MEDLINE	
OVID interface	
OVID interface	
OVID interface exp Rinderpest virus/ or exp Rinderpest/ or rinderpest*.ti,ab.	1,186
Limited to 2011–2022	162
CINAHL Complete	
EBSCOhost interface	
rinderpest* in Title OR rinderpest* in Abstract OR rinderpest* as a word in subject heading	14
Limited to 01/01/2011–12/31/2021	7
Web of Science Collection	
Databases searched simultaneously via Web of Science interface	
Science Citation Index- 1900-present	
Social Science Citation Index 1900-present	
Arts & Humanities Citation Index 1975-present	
Conference Proceedings Citation Index-Science 1990-present	
Conference Proceedings Citation Index-Social Science & Humanities 1900-present	
Book Citation Index-Science 2005-present	
Book Citation Index-Social Science & Humanities 2005-present	
Emerging Sources Citation Index 2005-present	
Current Chemical Reactions 1985-present	
Index Chemicus 1993-present	
TI = (rinderpest*) OR AB = (rinderpest*) OR TS = (rinderpest)	

Search terms	Results
Limited to 2011–2021	383
ABI INFORM<br>ProQuest interface<br>ab(rinderpest*) or ti(rinderpest*) or su(rinderpest*)<br>Limited to 2011–2021	32
Academic Search Ultimate<br>EBSCOhost interface<br>Title = rinderpest* OR Subject Terms = rinderpest* OR Abstract = rinderpest*<br>Limited to 01/01/2011–12/31/2021	176
NewsBank<br>NewsBank interface<br>Rinderpest in Headline OR rinderpest in Lead/First paragraph<br>Limited to 2011–2021	274

Appendix Table 2. Summary of studies involving rinderpest virus published between 2011 and 2021.

Study category	Summary	Lab location	Reference
Vaccine development/ Immunity	The crystal structure of MHC 1 N*01801 complexed to rinderpest derived peptide IPA was evaluated and analysis revealed that the MHC groove can assume different conformations to bind with the rinderpest viral peptide.	Beijing, China	*1*
	RPV was cultured by utilizing a vaccine strain of the virus and it was mutated based on potential C1 monoclonal antibody binding sites. After characterizing C1 binding sites, it was discovered that the deletion or mutation of these sites resulted in C1 not being able to bind to RPV.	Pirbright, United Kingdom *	*2*
	Cattle were vaccinated with either wild-type or two established PPRV vaccine strains to determine the degree of protection to which individuals vaccinated for PPR have against RPV. Only animals vaccinated with the wild-type PPRV were protected from RPV challenge. These individuals were also able to neutralize RPV-pseudotyped vesicular stomatitis virus.	Pirbright, United Kingdom*,‡	*3*
Diagnostics	The goal of this study was to develop a one-step multiplex reverse transcription PCR assay for the simultaneous detection of Rift Valley Fever Virus, Bluetongue Virus, RPV, and PPRV. Dual- Priming Oligonucleotide was used to develop the diagnostic test for the four viruses.	Anyang, Republic of Korea	*4*
	This study used a replication-defective vesicular stomatitis virus based pseudotyping system to measure neutralizing antibodies against RPV and PPR. This system does not require the use of live infectious viral materials and thus mitigates the risk of accidental exposure. Analysis revealed that individuals vaccinated for RPV also are protected against PPR infection. Individuals that were vaccinated against PPR had lower antibody titers than those who were naturally infected and in individuals infected with either PPR or RPV neutralizing responses were highest against the homologous virus. This indicates that retrospective analysis of serologic samples can be used to determine the pathogen to which an infected individual was exposed.	Pirbright, United Kingdom*,‡	*5*
	This study focuses on the development of a multiplex RT-PCR and automated microarray assay for the simultaneous detection of eight important cattle viruses: vesicular stomatitis virus, bluetongue virus, bovine viral diarrhea virus type 1 and 2, malignant catarrhal fever virus, bovine herpesvirus-1, parapox virus complex, and RPV.	Pirbright, United Kingdom*,‡	*6*
	Because of the request to destroy all RPV samples following eradication a new diagnostic method must be developed that does not rely on RPV as a positive material. Newcastle Disease with small RNA inserts based on RPV or PPV was used as a positive control for extraction, reverse transcription, and amplification.	Lelystad, Netherlands†	*7*
Enzyme activity	The V proteins of RPV, measles virus, PPR, and canine distemper were compared to determine which had the ability to block type 1 and type 2 interferon action. Analysis revealed that the V proteins of each morbillivirus could block type 1 interferon action but they had varying abilities to block type 2 interferon action which is correlated with the co-precipitation of STAT1 with the V protein. Further analysis revealed that all morbillivirus V proteins form a complex with Tyk2 and Jak2, two interferon-receptor-associated kinases.	Pirbright, UK*	*8*
	The enzymatic role of RPV V protein was investigated to determine how it blocks interferon signaling. Analysis revealed that the morbillivirus V proteins have at least three functions that inhibit interferon signaling, the binding of STAT1 also seen with P and W proteins) which enables the blockade of type 2 interferon signaling, the binding of STAT1 which requires the Vs domain and	Pirbright, United Kingdom*	*9*

Study category	Summary	Lab location	Reference
	part of the W domain, and the association with interferon receptor-associated kinases which also requires the Vs domain.		
	Partially purified recombinant RNA polymerase complex of RPV was used to show in vitro methylation of capped mRNA. Analysis revealed that the catalytic module for cap 0 methyl transferase activity is located in domain 3 of the L protein whereas domain 2 stabilizes the enzyme and increases catalytic efficiency. This provides support for the modular nation of the RPV L protein.	Bangalore, India§	*10*
	E. coli was used to express the RTPase domain of RPV to investigate the RTPase activity of L protein. Analysis revealed that L protein exhibits RTPase and NTPase activities and that it has a two-metal mechanism similar to the RTPase domain of other viruses.	Bangalore, India§	*11*
	E. coli was used to express the RTPase domain of RPV to investigate its enzymatic abilities. Analysis revealed that the L protein of RPV has RNA-dependent RNA polymerase, RTPase, Guanylyltransferase (GTase), and Methyltransferase activity in addition to pyrophosphatase (Ppase) and tripolyphosphatase (PPPase) activity.	Bangalore, India§	*12*
Genome sequencing	The B and L strains of RPV were sequenced to investigate host range and virulence factors. The stock B strain is pathogenic to cattle whereas the L strain is pathogenic to rabbits but not cattle and buffalo. Analysis revealed that differences in pathogenicity to cattle is caused by nt/aa substitution in P/C/V genes.	Tokyo, Japan*	*13*
	The LATC06 strain of RPV was sequenced and compared to other rinderpest viral strains. Analysis revealed that the functions of the LATC06 (Korea) and LA (Japan) strains of RPV are similar with regards to immunodominance in humoral immunity.	Anyang, Korea	*14*
	The genomes of three strains of RPV, L72, LA77, and LA96, were sequenced and analyzed to investigate their genetic variability. Analysis revealed that genetic variability occurs within the vaccine virus strain and that amino acid sequence similarity between Fusan and other strains was the lowest within the P, C, and V proteins. This indicated that the difference in pathogenicity of different strains may be Because of the V protein.	Anyang, Korea	*15*
	The LA-AKO strain of the RPV vaccine was sequenced. Analysis revealed that the bulk vaccine comprises mixed viral populations with minor mutations at the nucleotide level.	Ibaraki, Japan*,‡	*16*
	In preparation for the destruction of all RPV samples, the full genome sequence was determined of each distinct RPV sample housed at Pirbright. Analysis revealed that the African isolates form a single disparate clade as opposed to two separate clades and that the clade containing viruses developed in Korea were more similar to African viruses than Asian viruses.	Pirbright, United Kingdom*,‡	*17*

*Conducted in association with a current FAO-WOAH designated RHF
†Presented research conducted before 2011
‡Supported by the FAO-WOAH Joint Advisory Committee for Rinderpest.
§Rinderpest virus containing material (RVCM) was not used in these studies.

References

1. Li X, Liu J, Qi J, Gao F, Li Q, Li X, et al. Two distinct conformations of a rinderpest virus epitope presented by bovine major histocompatibility complex class I N*01801: a host strategy to present featured peptides. J Virol. 2011;85:6038–48. PubMed https://doi.org/10.1128/JVI.00030-11

2. Buczkowski H, Parida S, Bailey D, Barrett T, Banyard AC. A novel approach to generating morbillivirus vaccines: negatively marking the rinderpest vaccine. Vaccine. 2012;30:1927–35. PubMed https://doi.org/10.1016/j.vaccine.2012.01.029

3. Holzer B, Hodgson S, Logan N, Willett B, Baron MD. Protection of cattle against rinderpest by vaccination with wild-type but not attenuated strains of peste des petits ruminants virus. J Virol. 2016;90:5152–62. PubMed https://doi.org/10.1128/JVI.00040-16

4. Yeh JY, Lee JH, Seo HJ, Park JY, Moon JS, Cho IS, et al. Simultaneous detection of Rift Valley fever, bluetongue, rinderpest, and peste des petits ruminants viruses by a single-tube multiplex reverse transcriptase-PCR assay using a dual-priming oligonucleotide system. J Clin Microbial. 2011;49:1389–94.

5. Logan N, Dundon WG, Diallo A, Baron MD, James Nyarobi M, Cleaveland S, et al. Enhanced immunosurveillance for animal morbilliviruses using vesicular stomatitis virus (VSV) pseudotypes. Vaccine. 2016;34:5736–43. PubMed https://doi.org/10.1016/j.vaccine.2016.10.010

6. Lung O, Furukawa-Stoffer T, Burton Hughes K, Pasick J, King DP, Hodko D. Multiplex RT-PCR and automated microarray for detection of eight bovine viruses. Transbound Emerg Dis. 2017;64:1929–34. PubMed https://doi.org/10.1111/tbed.12591

7. van Rijn PA, Boonstra J, van Gennip HGP. Recombinant Newcastle disease viruses with targets for PCR diagnostics for rinderpest and peste des petits ruminants. J Virol Methods. 2018;259:50–3. PubMed https://doi.org/10.1016/j.jviromet.2018.06.007

8. Chinnakannan SK, Nanda SK, Baron MD. Morbillivirus V proteins exhibit multiple mechanisms to block type 1 and type 2 interferon signalling pathways. PLoS One. 2013;8:e57063. PubMed https://doi.org/10.1371/journal.pone.0057063

9. Chinnakannan SK, Holzer B, Bernardo BS, Nanda SK, Baron MD. Different functions of the common P/V/W and V-specific domains of rinderpest virus V protein in blocking IFN signalling. J Gen Virol. 2014;95:44–51. PubMed https://doi.org/10.1099/vir.0.056739-0

10. Gopinath M, Shaila MS. Evidence for N^7 guanine methyl transferase activity encoded within the modular domain of RNA-dependent RNA polymerase L of a morbillivirus. Virus Genes. 2015;51:356–60. PubMed https://doi.org/10.1007/s11262-015-1252-3

11. Singh PK, Ratnam N, Narayanarao KB, Bugatha H, Karande AA, Melkote Subbarao S. A carboxy terminal domain of the L protein of rinderpest virus possesses RNA triphosphatase activity—the first enzyme in the viral mRNA capping pathway. Biochem Biophys Res Commun. 2015;464:629–34. PubMed https://doi.org/10.1016/j.bbrc.2015.07.026

12. Singh PK, Subbarao SM. The RNA triphosphatase domain of L protein of rinderpest virus exhibits pyrophosphatase and tripolyphosphatase activities. Virus Genes. 2016;52:743–7. PubMed https://doi.org/10.1007/s11262-016-1353-7

13. Fukai K, Morioka K, Sakamoto K, Yoshida K. Characterization of the complete genomic sequence of the rinderpest virus Fusan strain cattle type, which is the most classical isolate in Asia and comparison with its lapinized strain. Virus Genes. 2011;43:249–53. PubMed https://doi.org/10.1007/s11262-011-0630-8

14. Yeh JY, Kwoen CH, Jeong W, Jeoung HY, Lee HS, An DJ. Genetic characterization of the Korean LATC06 rinderpest vaccine strain. Virus Genes. 2011;42:71–5. PubMed https://doi.org/10.1007/s11262-010-0543-y

15. Jeoung HY, Lee MH, Yeh JY, Lim JA, Lim SI, Oem JK, et al. Complete genome analysis of three live attenuated rinderpest virus vaccine strains derived through serial passages in different culture systems. J Virol. 2012;86:13115–6. PubMed https://doi.org/10.1128/JVI.02362-12

16. Takamatsu H, Terui K, Kokuho T. Complete genome sequence of Japanese vaccine strain LA-AKO of rinderpest virus. Genome Announc. 2015;3:e00976–15. PubMed https://doi.org/10.1128/genomeA.00976-15

17. King S, Rajko-Nenow P, Ropiak HM, Ribeca P, Batten C, Baron MD. Full genome sequencing of archived wild type and vaccine rinderpest virus isolates prior to their destruction. Sci Rep. 2020;10:6563. PubMed https://doi.org/10.1038/s41598-020-63707-z
